# Baicalin Protects Mice from Lethal Infection by Enterohemorrhagic *Escherichia coli*

**DOI:** 10.3389/fmicb.2017.00395

**Published:** 2017-03-09

**Authors:** Yong Zhang, Zhimin Qi, Yan Liu, Wenqi He, Cheng Yang, Quan Wang, Jing Dong, Xuming Deng

**Affiliations:** ^1^The First Hospital and Institute of Infection and Immunity, Jilin UniversityChangchun, China; ^2^Key Laboratory of Zoonosis, Ministry of Education, Institute of Zoonosis, College of Veterinary Medicine, Jilin UniversityChangchun, China; ^3^High Throughput Molecular Drug Discovery Center, Tianjin International Joint Academy of Biotechnology and MedicineTianjin, China; ^4^Yangtze River Fisheries Research Institute, Chinese Academy of Fishery SciencesWuhan, China

**Keywords:** Shiga-like toxin, *Escherichia coli* (STEC) O157:H7, baicalin, infection, antibiotics

## Abstract

Shiga-like toxin-producing *Escherichia coli* (STEC) O157:H7 poses grave challenges to public health by its ability to cause severe colonic diseases and renal failure in both human and animals. Shiga-like toxins are the major pathogenic factor for some highly virulent *E. coli* expecially Shiga-like toxin 2. Conventional treatments such as antibiotics can facilitate the release of the toxin thus potentially exacerbate the diseases. Small molecule inhibitors and antibodies capable of neutralizing the toxins are the two major venues for the development of therapeutics against enterohemorrhagic serotype *E. coli* infection. While promising and potentially effective at clinical settings, these approaches need to overcome obstacles such as the limited routes of administration, responses from the host immune system, which are known to differ greatly among individuals. Our previous studies demonstrate that Baicalin (BAI), a flavonoid compound isolated from *Scutellaria baicalensis* protects against rStx2-induced cell cytotoxicity and also protects mice from lethal rStx2 challenges by inducing Stx2 to form inactive oligomers. In this manuscript, we present some exciting work showing that baicalin is an effective agent for therapeutic treatment of STEC O157:H7 infection.

## Introduction

Infection by the enterohemorrhagic serotype *Escherichia coli* (EHEC) O157:H7 leads to clinical symptoms ranging from watery or bloody diarrhea to the life-threatening hemolytic-uremic syndrome (HUS) associated with kidney failure in both human and animal hosts, such as greyhound dogs (Gerber et al., 2002). Cattle and sheep are among the most important reservoir hosts of this pathogen (Raya et al., 2006; Jaros et al., 2016). EHEC infection is a leading cause of these diseases. For example, in the United States, about 36% of the 265,000 STEC infections are caused by strain O157:H7 (Zama and Fariza, 2014). Infection by *E. coli* O157:H7 often occurs following the consumption of contaminated food or drink. The bacterium is highly virulent; a low infectious dose in the range of 10 to 100 colony-forming units (CFU) is sufficient to cause successful infections (Buchanan and Edelson, 1996). Shiga toxins (Stx) expressing from a prophage are considered the single most important virulence factor of this group of pathogenic *E. coli* (O’Brien et al., 1992). Strains expressing Stx2 alone are more virulent than those that express both Stx1 and Stx2 (Ostroff et al., 1989). Furthermore, Stx2 is about 1000 times more toxic than Stx1 (Louise and Obrig, 1995). Thus, Stx2 plays a dominant role in the pathogenicity of STEC.

Treating infections caused by *E. coli* O157:H7 has been historically challenging. The usefulness of traditional antibiotics in the treatment of HUS has been a subject of debate. Because of the potential induction of the production and release of the Stx by antimicrobial agents, chemotherapy is not recommended for patients with diarrhea caused by O157:H7 or *E. coli* elaborating similar toxins (Wong et al., 2000). For example, antibiotics of the quinolone family stimulate Stx production by *E. coli* O157:H7, the transfer of the prophage and more deaths (Zhang et al., 2000). As a result, several alternative therapeutic strategies have been developed. Among these, the use of analogs of Gb3, the receptor of Stx to block the recognition of the toxin by the cells (Nishikawa et al., 2005) and monoclonal antibodies that specifically neutralize the toxins (Yamagami et al., 2001) has gained considerable success in animal models. Similarly, a recent study showed that Retro-2^cycl^, a synthetic inhibitor for retrograde trafficking of mammalian cells is effective in protecting mice from lethal infections by *E. coli* O104:H4 (Secher et al., 2015). While promising and potentially effective at clinical settings, these approaches need to overcome obstacles such as the limited routes of administration, responses from the host immune system, which are known to differ greatly among individuals. For agents such as Retro-2^cycl^ that directly targets an essential host cellular process, its potential (detrimental) effects warrant further evaluation. These limitations have urged the development of novel effective therapeutics that is low-cost, easy to use and known of low toxicity.

Our previous studies demonstrate that Baicalin (BAI), a flavonoid compound isolated from *Scutellaria baicalensis* protects mice from lethal Stx2 challenges by inducing Stx2 to form inactive oligomers (Dong et al., 2015). Here, we extended our study on the protective effects of BAI in the treatment of O157:H7 infections in both tissue culture and a mouse model that mimics clinical outcomes. Our results show that BAI is a potentially useful compound in treating O157:H7 infections caused by its natural route of infection.

## Materials and Methods

Mitomycin C (MMC) treatment is known to induce the production of Stx toxins, particularly Stx2, leading to exacerbation of the disease symptoms (Fujii et al., 1994); we thus first determined the effects of this agent on the production of Stxs by O157:H7 strain EDL933 (Miranda et al., 2004). A sub-inhibitory concentration (100 ng/ml) of MMC was added to cultures of strain EDL933 for 14 h; bacterium-free culture supernatant obtained from the cultures was incubated with Hela cells.

We next evaluated the protection effects of BAI on mice infected with O157:H7 strain EDL933 (ATCC 43895). All animal studies were performed according to the regulations for the Administration of Affairs Concerning Experiments Animals (1988.11). The experimental protocols were approved and supervised by the Institutional Animal Care and Use Committee of Jilin University.

BALB/c mice of 8-week age weighing between 16 and 18 g obtained from the Experimental Animal Center of Jilin University (Changchun, China) were given streptomycin (5 g/L in water) *ad libitum* for 3 days to disrupt their normal intestinal flora (Fujii et al., 1994). On day 3, mice were starved for 6 h prior to infection. For mortality study, 15 mice in each group were challenged with 1.5 × 10^8^ cfu/200μl bacterial suspension through orally administration simultaneously with a single dose of MMC (2.5 mg/kg of body weight) by intraperitoneal injection. As controls, mice not treated with MMC were similarly inoculated with identical bacterial suspensions. At 24 h post–inoculation, BAI was given to the infected mice through the oral route at 100 mg per kg of body weight, and then at 8-h intervals for an additional 5 days (Supplementary Table S1).

We also determined the ability of BAI in protecting against the weight loss caused by sub-lethal infections by EDL933. BAI alone at a dose as high as 100 mg/kg did not detectably affect mouse body weight over a 10-day period (Dong et al., 2015). In our experiments here, mice inoculated with PBS or with a single dose of O157:H7 (7.5 × 10^7^ per mouse) were monitored for body weight at 24-h intervals. BAI was given at 100 mg/kg 24-h after infection and then at 8-h intervals for 10 days (Supplementary Table S2).

Next, we evaluated the effects of BAI treatment in alleviating renal injuries caused by O157:H7 infection. Mice infected with 7.5 × 10^7^ cfu together with MMC were either treated with BAI as described above or with PBS. At 6-day post-infection, kidneys from mice of different experimental groups were sectioned for pathological examination (Supplementary Table S2).

### Ethics Statement

All animal studies were performed according to the Regulations for the Administration of Affairs Concerning Experiments Animals (1988.11). The experimental protocols (Protocol number: 20140623006) were approved and supervised by the Institutional Animal Care and Use Committee (IACUC) of Jilin University.

The source of O157:H7 strain EDL933 is ATCC 43895.

## Result

### BAI Protects Cells against Toxins Released by EHEC Strain EDL933

**FIGURE 1:**
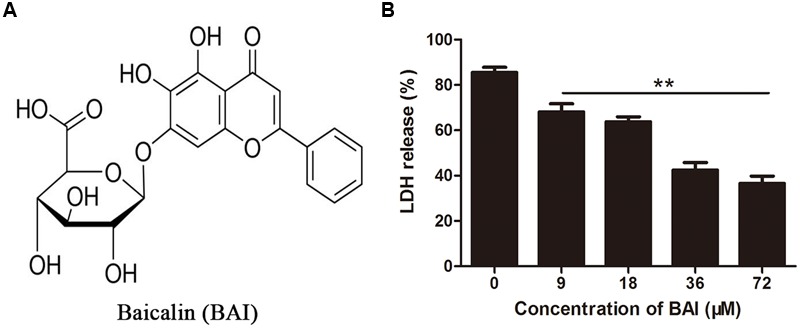
**Baicalin protects cells against toxins released by EHEC strain EDL933. (A)** Chemical structure of Baicalin. **(B)** Inhibition of cell death by baicalin. Supernatant of EHEC EDL933 was added into Hela cells treated with indicated concentrations of baicalin for 36 h. Cell viability was evaluated by measuring extracellular LDH 36 h after adding the supernatant. All data represent the mean and standard error of three independent experiments. ^∗∗^*p* < 0.01.

*In vitro* protection assay showed that LDH released by cells receiving culture supernatant from MMC-treated bacteria was significantly higher than that from untreated cultures, indicating that MMC induced the release of Stx toxins. Importantly, inclusion of BAI (**Figure 1A**) in the culture supernatant protected Hela cells from Stx-induced cell damage. Significant protection was achieved when BAI was used at 9 μM and the maximal protection was observed at 72 μM under our experimental conditions (**Figure 1B**).

### BAI Protects Mice from EHEC Strain EDL933 Infection

**FIGURE 2:**
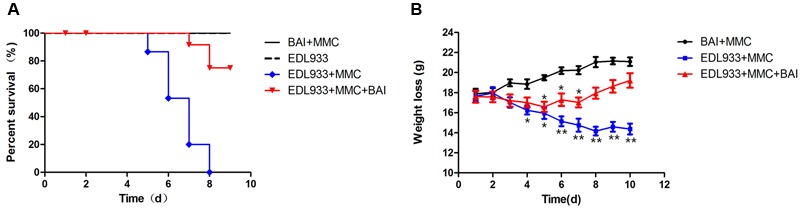
**Baicalin protects mice against lethal infection caused by EHEC strain EDL933. (A)** Groups (*n* = 15) of mice infected with EDL933 via the oral route together with MMC injection were treated with saline solution or with BAI 24 h post-infection. The survival of the animals was monitored for 10 days. **(B)** The effects of BAI on body weight loss caused by sub-lethal doses of EDL933. Groups (*n* = 10) of mice infected with EDL933 as described in **(A)** were administered with BAI or PBS. The body weight was monitored daily for 10 days. Similar results were obtained from more than three independent experiments.

Mice protection effect of BAI was evaluated and a single challenge of O157:H7 EDL933 without MMC caused no animal death within 15 days. In contrast, a single inoculation of the bacteria together with MMC caused 100% animal deaths within 8 days (**Figure 2A**). In groups that received BAI, the death rate at day 8 was about 20% (**Figure 2A**), indicating that this compound provides approximately 80% protection to O157:H7-infected mice.

Body weights of the tested mice were monitored and our results showed that infected mice not receiving BAI exhibited decreased food and water intake; body weight loss became apparent on day 4, and reached the lowest point at day 8 (**Figure 2B**). BAI treatment delayed the occurrence of body weight loss for 48 h; Furthermore, the loss was significantly less severe throughout the experiment duration (**Figure 2B**). As expected, in line with their normal health status, the body weight of mice receiving PBS increased throughout the experimental duration (**Figure 2B**). Our choice of the 24-h treatment window after infection was based on the comparison of several time points including 12, 24, 36, and 48 (**Figure 2A**). The 24-h provided the best protection and thus was used for all the relevant experiments.

### Quantitative Analysis of Histopathology Changes Showed Alleviated Pathologies of BAI Treatment

**FIGURE 3:**
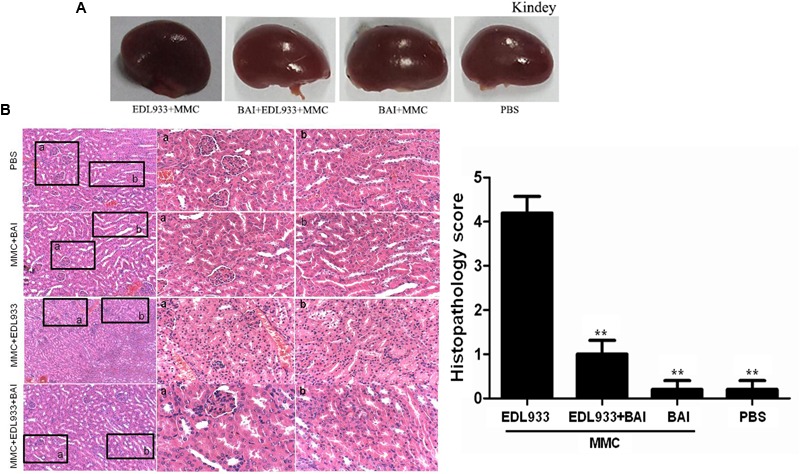
**Kidneys tissue and their histopathological examination by HE staining. (A)** Comparison of kidneys tissue from differently treated mice. Note that kidneys from BAI-treated mice exhibited lower pathological abnormalities. **(B)** Histopathological examination of kidney tissue by HE staining. PBS and MMC+BAI group displayed standard kidney histopathology. a: complete glomerulus architecture, b: regular nuclear arrangement in renal tubule. EDL933 infection group space between the glomerulus and Bowman’s capsule; b: loss of brush border in the renal tubule, along with cytoplasmic attending and loss of the epithelial cells; loss of polarity of tubular cells and degeneration in the renal tubule. BAI administration improved kidney histopathology following EDL933 treatment. a: complete Bowman’s capsule and glomerulus architecture; b: renal tubule with distributed brush border; Regular nuclear arrangement, and well-development renal tubule. Histopathology scoring of renal glomerular degeneration and renal tubule necrosis by a veterinary pathologist: grade 0: no injury; grade 1: minimal injury with less than 10% of cells exhibiting degeneration or necrosis; grade 2: mild injury involving 10–25% of cells; grade 3: moderate injury involving 25–40% of cells; grade 4: marked injury involving 40–50% of cells; grade 5: severe injury involving greater than 50% of cells. *N* = 5, ^∗∗^*p* < 0.01 vs. EDL933 infection group.

Histopathology changes of renal glomerular degeneration and renal tubule necrosis damages were quantified by a histopathologist in a blind manner. Our results showed that damages such as swelling, discoloration and necrosis were severe in kidney tissues from infected mice not receiving BAI (**Figure 3A**), while histopathological examination of kidney tissue of BAI treatment group (Gu et al., 2016) indicated that BAI treatment significantly reduced renal damage in mice (**Figure 3B**).

**FIGURE 4:**
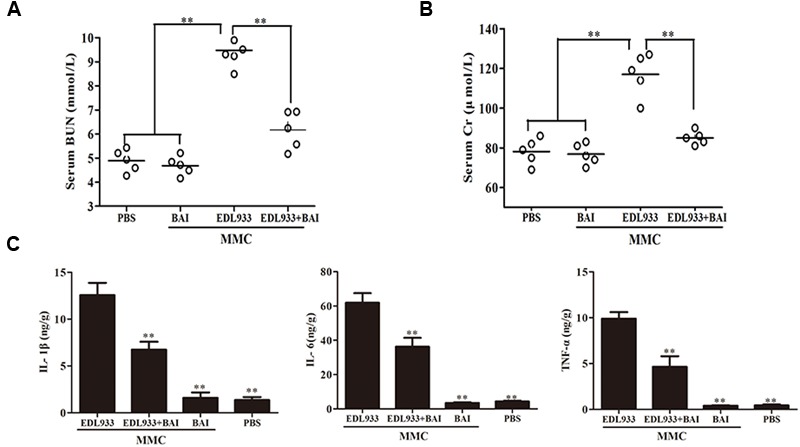
**Baicalin alleviated the pathologies induced by EDL933 infection. (A,B)** The effects of baicalin on blood urea nitrogen (BUN) **(A)** and creatinine (Cr) **(B)**. Induced by EDL933 infection. The blood of relevant mice groups was evaluated for BUN and Cr at 6 days after treatment. Each circle represents the BUN or Cr level from one mouse, *n* = 5. **(C)** Baicalin reduced the production of several cytokines induced by EDL933. Kidney tissues from relevant mice were measured for cytokines by ELISA. Note the reduction of cytokine production in infected mice treated with baicalin. ^∗∗^*p* < 0.01.

Serological index and the release of pro-inflammatory of the kidneys were tested. Six days after infection, the level of blood urea nitrogen (BUN) in infected mice was 9.508 ± 0.414 mM; BAI treatment reduced it to 6.143 ± 0.453 mM. In fact, the level in treated mice was close to that of uninfected mice, which was 4.675 ± 0.226 mM (**Figure 4A**). BAI treatment similarly reduced the presence of creatinine (Cr) in the serum of infected mice (**Figure 4B**). In agreement with these results, BAI treatment significantly lowered the secretion of several cytokines such as IL-1β, IL-6 and TNF-α associated with kidney damages caused by O157:H7 infection (**Figure 4C**). Taken together, these results indicate that BAI effectively protect the kidney from the damage caused by O157:H7 infection.

## Discussion

Because of the high mortality and morbidity associated with infections caused by O157:H7, extensive multidisciplinary efforts have been invested to identify effective treatments. For example, vaccines (Szu and Ahmed, 2014) and immunotherapies have been described (Yamagami et al., 2001). The use of Stx receptor analogs (Nishikawa et al., 2002, 2005) and antibodies specific to the toxins has also been reported. A strategy that combined antibiotics and antibody specific for the Stx appeared effective in *in vitro* experiments (Skinner et al., 2015). The small molecule Retro-2^cycl^ that functions by inhibiting host retrograde trafficking is effective against infections caused by the EHEC strain O104:H4 in a mouse disease model (Secher et al., 2015).

Enterohemorrhagic serotype *E. coli* contains various virulence factors, among which stx2 is the plays important role in the pathogenicity of STEC O157 strain by neutralize the toxicity of stx2, STEC O157: H7 becomes ordinary *E. coli* and clearance by the organism (Nathan, 2002; Mombelli et al., 2011; Wiersinga, 2011). O104 may have the same mechanism as well. Although the role of baicalin inhibiting the activity of rStx2 *in vitro* and *in vivo* (Dong et al., 2015), the key virulence factor of EHEC strains, was clarified in our previous study, the effect of baicalin against EHEC infection was still unclear. There are several virulence factors and other pathogenic factors secreted by EHEC strains. Whether baicalin could still be a useful agent against EHEC infection in such complicated condition was need to be clarified. Furthermore, the toxin in our previous study was a recombinant production, the activity, dosage and the infective mode was completely different from natural infection by EHEC strains. Thus, we designed experiments in this study to further demonstrate the protective effect of baicalin against EHEC infection. Our demonstration of the effectiveness of BAI against O157:H7 infection in a post-infection manner, adds another useful avenue to meet the challenges posed by Stx-producing pathogens. Although the exact post-infection treatment window for human remains to be determined, the 24-h window found in mice should provide the reference for the time frame necessary for good clinical outcomes, particularly for patients suspected of consuming EHEC contaminated food. Comparing to therapeutics such as toxin-specific antibodies and agents that inhibit important host cellular processes, the advantages of BAI lie in its low toxicity, high stability that allows convenient transportation and storage. Like all compounds that proved to be effective in animal model experiments, the next challenge is to determine whether BAI is effective in the treatment of infections caused by Stx-producing *E. coli* either by itself or in combination with other agents.

## Author Contributions

JD and XD conceived the project. YZ, ZQ, JD, and XD designed the experiments. YZ, ZQ, and YL performed the research. YZ, JD, and XD wrote the paper and all authors made editorial input.

## Conflict of Interest Statement

The authors declare that the research was conducted in the absence of any commercial or financial relationships that could be construed as a potential conflict of interest.
