# Supplementary material for: Baicalin Protects Mice from Lethal Infection by Enterohemorrhagic Escherichia coli
Source: Front Microbiol. 2017 Mar 9;8:395. doi: 10.3389/fmicb.2017.00395 (PMC5343029; doi:10.3389/fmicb.2017.00395)
Supplement: Supplementary file 1 [file Table_1.doc]

| **Group** | **Bacteria concentration**  **（cfu/ml）** | **drug administration*** | **MMC dose (mg/kg)** | **BAI dose**  **(mg/kg)** |
| --- | --- | --- | --- | --- |
| BAI+MMC^#^ | NA | 8h intervals for 5 days. | 2.5 | 100 |
| EDL933 | 7.5x10^8^ | NA | NA | NA |
| EDL933+MMC^#^ | 7.5x10^8^ | NA | 2.5 | NA |
| EDL933+MMC^#^+BAI | 7.5x10^8^ | 8h intervals for 5 days. | 2.5 | 100 |

**Table S1** The treatment of different groups for survival experiments

# MMC was administrated by injection at the time of infection with EHEC.

* BAI was administrated 24h post infection.

**Table S2** The treatment of different groups for other experiments

| **Group** | **Bacteria concentration**  **（cfu/ml）** | **drug administration*** | **MMC dose (mg/kg)** | **BAI dose**  **(mg/kg)** |
| --- | --- | --- | --- | --- |
| BAI+MMC^#^ | NA | 8h intervals for 10 days^a^.  8h intervals for 6 days^b^. | 2.5 | 100 |
| EDL933+MMC^#^ | 3.75x10^8^ | NA | 2.5 | NA |
| EDL933+MMC^#^+BAI | 3.75x10^8^ | 8h intervals for 10 days^a^.  8h intervals for 6 days^b^. | 2.5 | 100 |

# MMC was administrated by injection at the time of infection with EHEC.

* BAI was administrated 24h post infection.

a for weight loss experiment.

b for treatment experiment in alleviating renal injuries.
